# Drug Interaction of Dasatinib with Thymoquinone: A Pharmacokinetic Study in Rats

**DOI:** 10.7150/ijms.109707

**Published:** 2025-09-03

**Authors:** Ajaz Ahmad, Mohammad Raish, Khalid M. Alkharfy, Yousef A Bin Jardan, Abdul Ahad, Mohd Abul Kalam, Muzaffer Iqbal, Ibrahim A. Abdelrahman, Naushad Ali, Ali Akhtar, Fahad I Al-Jenoobi

**Affiliations:** 1 Department of Clinical Pharmacy, College of Pharmacy, King Saud University, Riyadh 11451, Saudi Arabia.; 2 Department of Pharmaceutics, College of Pharmacy, King Saud University, Riyadh 11451, Saudi Arabia.; 3 Department of Pharmaceutical Chemistry, College of Pharmacy, King Saud University, Riyadh 11451, Saudi Arabia.; 4 Quality Assurance Unit, College of Pharmacy, King Saud University, Riyadh 11451, Saudi Arabia.

**Keywords:** Dasatinib, thymoquinone, pharmacokinetic, interaction, CYP3A2, Pgp, BCPR.

## Abstract

Dasatinib (DAS), a multi-kinase inhibitor targeting Src and BCR-ABL families, is approved for Ph+ acute lymphoblastic leukemia (ALL) and chronic lymphocytic leukemia (CML). Its metabolism by CYP3A4 and transported by efflux pump P-glycoprotein (Pgp) and breast cancer resistance protein (BCRP) render it susceptible to drug, food, and herbal interactions. This study investigated the potential impact of thymoquinone (TQ) co-administration on dasatinib pharmacokinetics (PK) and the subsequent risk of altered efficacy or toxicity. Wistar rats were pretreated with a daily oral dose of TQ (40 mg/kg) for one week before receiving a single oral dose of DAS (25 mg/kg). Blood samples were collected at different time points, and plasma concentrations of DAS were measured using UPLC-MS/MS. A non-compartmental analysis was applied to calculate the PK parameters of DAS. Additionally, the impact of TQ treatment on hepatic and intestinal protein expressions of CYP3A4, Pgp and BCRP were investigated using Western blot analysis. TQ pretreatment significantly altered the disposition of DAS in animals as compared to untreated animals. More specifically, a substantial increase in DAS Cmax (213.26%), AUC_0-t_ (166.53%), AUMC_0-∞_ (0.34%), K*el* (93.85%) and Tmax (83.33%) were observed (*p<0.05*). In addition, a significant reduction in DAS Vd (67.70%) and clearance CL (36.35%) of were evident in the TQ treatment group (*p<0.05*). Furthermore, TQ pretreatment inhibited CYP3A4 and Pgp and BCRP1 in the liver and lumen tissues. TQ pretreatment significantly alters the disposition of DAS in rats. This is likely due to an increase in DAS bioavailability *via* a modulation in the protein expressions of CYP3A4 and Pgp and BCRP1 in the liver and lumen tissues. Thus, the concurrent intake of TQ-containing products with DAS can lead to a serious interaction. Further clinical studies are warranted to evaluate the clinical impact of such observations.

## Introduction

**Figure 1:**
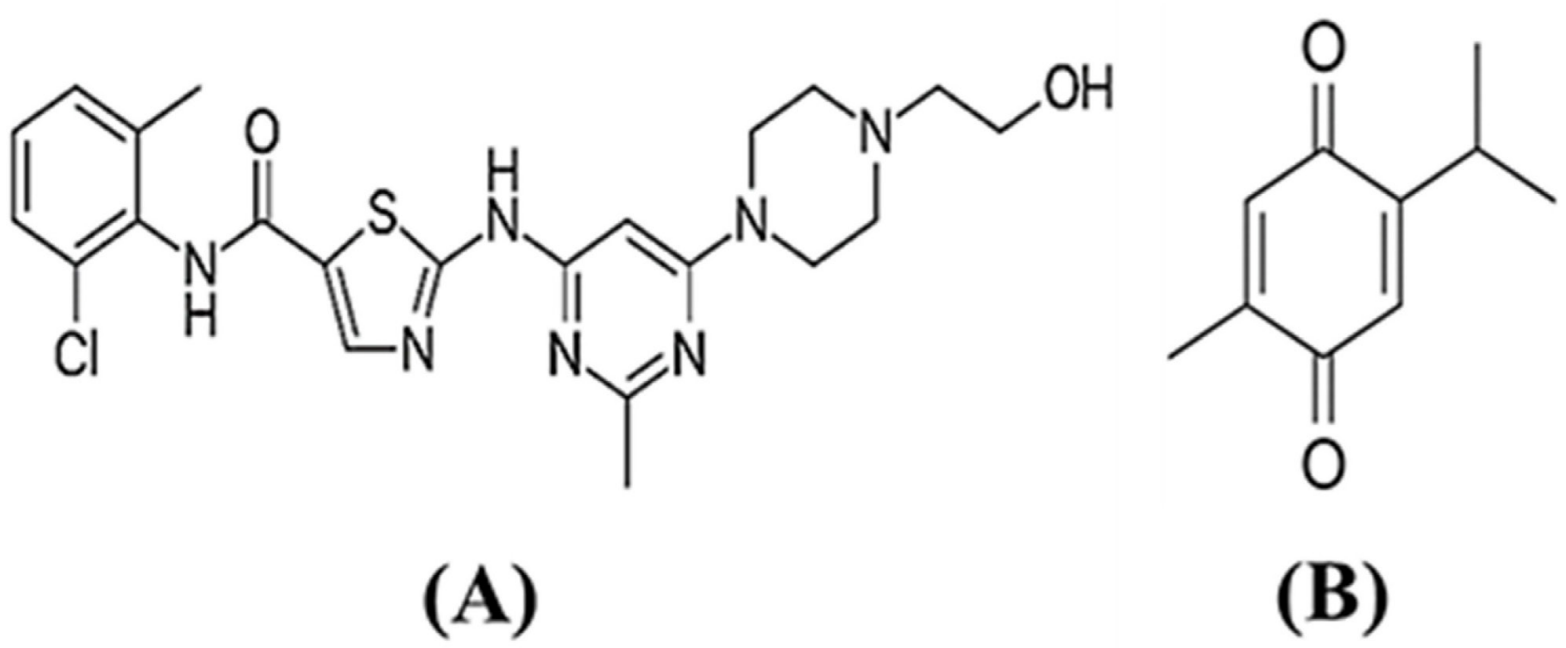
(A) Chemical structure of dasatinib 2-amino-1,3-thiazole-5-carboxylic acid and at position 6 by a 4-(2-hydroxyethyl)piperazin-1-yl. (B) TQ chemical structure (2-Methyl-5-(propan-2-yl)cyclohexa-2,5-diene-1,4-dione).

The tyrosine kinase (TK), a pivotal regulator within the cell cycle signaling pathways, has been linked to controlling proliferation, apoptosis, cell division, and metabolism 1. Thus, TK pathway dysfunction (overexpression or somatic mutation) causes an array of human diseases, especially cancers 2. In recent times, tyrosine kinase inhibitors (TKIs) have become among the most renowned pathway-directed chemotherapy medications for treating several types of cancer and exhibit fewer adverse reactions than conventional chemotherapy regimens 3, 4. Dasatinib (DAS, Figure 1A) is a potent orally available TKI, for PDGFR-β5, BCR-ABL, SRC family kinases, and c-KIT. DAS has been utilized in the treatment of both acute lymphoblastic leukemia (CML) and acute lymphocytic leukemia (ALL) 5-7. DAS is broadly absorbed from the intestine and is primarily metabolized in the liver 8-11. Unambiguously, DAS undergoes CYP3A4 facilitated metabolism and the key metabolites are M4, M5, M6, M20 and M24 7, 12, 13. M4 is formed due to N-dealkylation by CYP3A4, while M20 and M24 are formed by hydroxylation 9, 10. Not only DAS serves as a substrate for CYP3A4, but it also a target for efflux transporters such as P-glycoprotein (Pgp) and Breast Cancer Resistance Protein (BCRP) 14-19. Treatment with DAS has been linked to some serious toxicities, including heart failure, peripheral arterial occlusive disease (PAOD), QT prolongation, hypertension, reactivation of Hepatitis B virus (HBV), thrombosis, hyperglycemia, myelosuppression, hypocalcemia, cytopenia, hyperlipidemia, pulmonary hypertension, pneumonitis, and pleural effusion 20, 21.

Thymoquinone (TQ, Figure 1) is a bioactive polyphenol present in *Nigella sativa* (black seed) 22, 23. Because of its many pharmacological properties, TQ is commonly used in food and nutraceutical products. It has demonstrated antineoplastic, antioxidant, anti-inflammatory, and antidiabetic, activities 23, 24. Drug-drug interactions (DDIs) are increasingly being recognized as significant clinical events which can be detrimental 25-27. Several studies have suggested that TQ functions as an inhibitor of CYP3A4 23, 28-30 and Pgp and BCRP 31. Therefore, possible interactions with TQ are more likely to occur with drugs that undergo a high first pass metabolism. Both the liver and lumen mucosa contain the CYP3A4 enzyme. After absorption by the mucosa, vulnerable drugs may be pumped back into the intestine lumen by Pgp and BCRP, and metabolized by CYP3A4. Consequently, it's crucial to carefully assess the safety implications of using TQ products concurrently with DAS. Concerns about such interactions have been raised due to the consumption of products containing TQ alongside with prescribed medications, which could potentially alter the pharmacokinetics (PK) and the pharmacodynamics (PD) of interacting drugs 31, 32. Therefore, the aim of this study is to investigate the potential PK interaction between DAS and TQ; also, to explore the possible underlying mechanism of such consequence, if any.

## Materials and Methods

### Chemicals

Ammonium acetate, Ibrutinib (IRB), TQ, DAS were purchased from Sigma-Aldrich (St. Louis, MO, USA). Methanol, acetonitrile, and formic acid (HPLC grade) were procured from BDH Chemicals (Poole, UK). Anti- Pgp (sc-55510), CYP3A2 (sc-271033), BCPR (sc-58222) and β-actin (sc-47778) antibodies were obtained Santa Cruz Biotechology (Dallas, TX, USA).

### Animals and pharmacokinetic studies

The adult male Wistar rats (204-226 g) were procured from the College of Pharmacy Animal Care and Use Facility, King Saud University, Riyadh, Saudi Arabia. The animals were hold in polycarbonate rat cages, with 12h light/dark cycle at 25 ± 2 ºC. All groups were allowed *ad libitum* before the study. This study was conducted following the ethical guidelines approved by the facility and the study was approved by the King Saud University College of Pharmacy Research Ethics Committee (KSU-SE-21-58). Overnight fasted animals (n=24) were divided into four groups (n=6 per group). Group I (normal controls) and Group II were administered normal saline by oral gavage for seven consecutive days. On the seventh day, Group II received a single dose of DAS (25 mg/kg, p.o.) via oral gavage. Group III received TQ (40 mg/kg, p.o.) via oral gavage for seven days, and two hours after the final TQ dose, DAS (25 mg/kg, p.o.) was administered by oral gavage. Group IV received TQ (40 mg/kg/day) by oral gavage for seven consecutive days. For the PK study, blood samples were collected via a tail vein in heparinized vacutainers at various time intervals (i.e., 0, 0.5, 1, 2, 4, 6, 8, 10, 12 h post DAS dosing). The collected blood was centrifuged at 3000 ×g for 10 minutes to separate the plasma, which was then collected and stored in Eppendorf tubes for UHPLC-MS/MS analysis. After completion of the study, the animals were euthanized, and liver and lumen tissues were collected for Western blot analysis. DAS concentrations in plasma samples were determined using an UHPLC-MS/MS method according to a previously described method 17. Briefly, DAS and ibrutinib (IBR;(internal standard, IS) were separated on an Acquity BEH C18 column. Prior to sample analysis, the method underwent validation for precision and accuracy 33. A non-compartmental analysis was empoyed to calculate the PK parameters of DAS using the PK Solver software (version 1.01).

### Western blotting

The liver and intestine tissues were homogenized in RIPA lysis buffer and incubated for 30 minutes, followed by centrifugation at 10,000 rpm for 20 minutes at 4 °C. The supernatants were then collected and stored at -20 °C. Total protein content was determined using the bicinchoninic acid (BCA) assay. Protein expression of CYP3A2, Pgp, and BCRP proteins were analyzed according to previously published methods 15, 17, 34, 35.

### Statistical analysis

All data are expressed as the mean ± SEM. The pharmacokinetic and protein expression data were compared using one-way analysis of variance followed by Dunnett's test and a p value of *≤0.05* was used for statistical significance.

## Results

**Figure 2:**
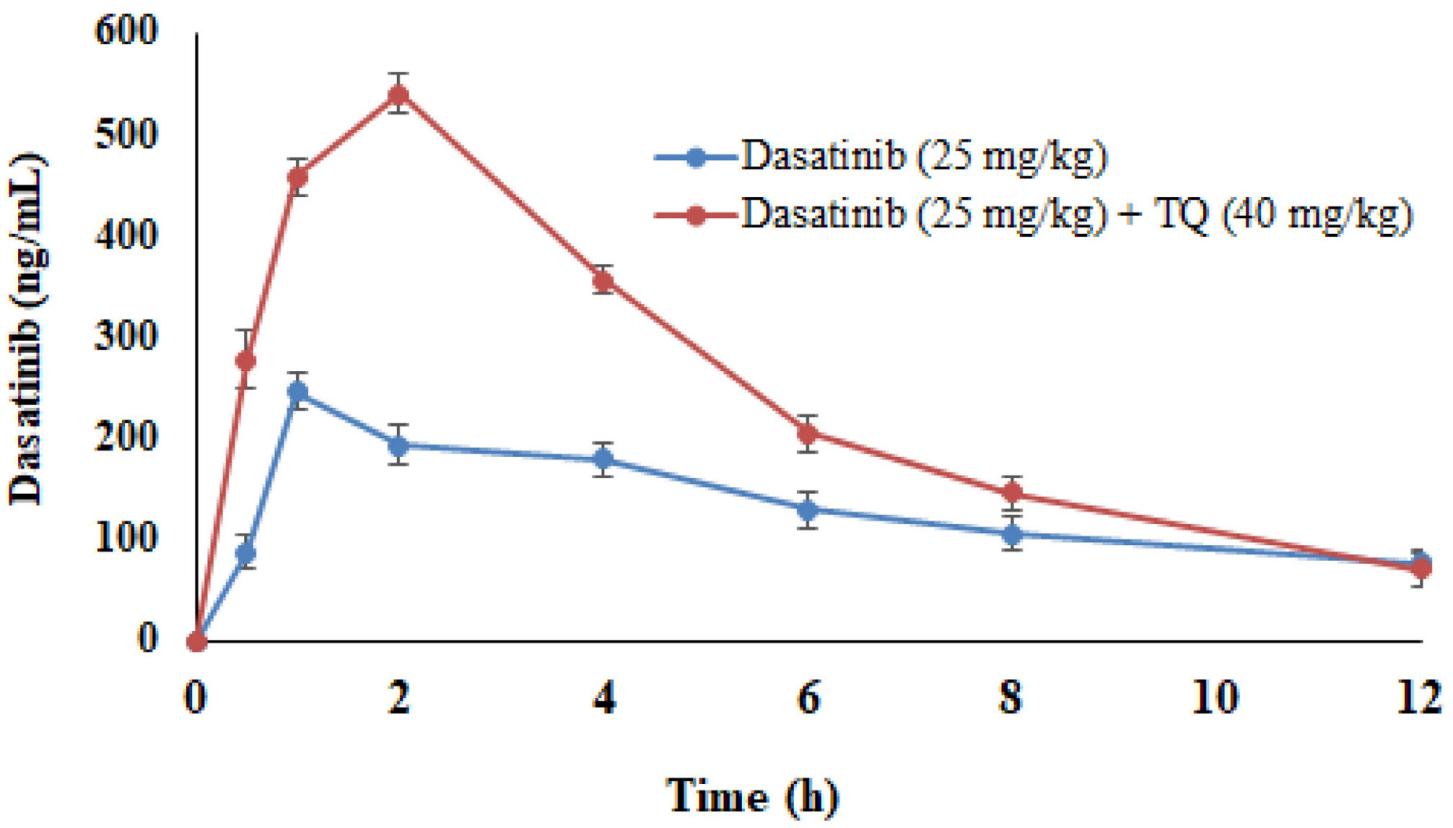
Dasatinib (DAS) plasma concentrations with and without TQ co-administration in rats. Data represent mean ± SEM. *p<0.05 vs. controls; #*p<0.05* vs. the DAS group (n= 6 per group).

Table 1 provides the PK parameters of DAS following an oral administration in rats. TQ pretreatment resulted in a substantial increase in DAS Cmax (213.26%), AUC_0-t_ (166.53%), AUMC_0-∞_ (0.34%), Kel constant (93.85%) and Tmax (83.33%), while the MRT (49.12%), T_1/2_ (11.56%), Vd (67.70%) and CL (36.35%) were significantly decreased as compared to the control group (*p<0.05*). Figure 2 demonstrated the plasma concentration profiles of DAS alone and with TQ pretreatment.

**Figure 3:**
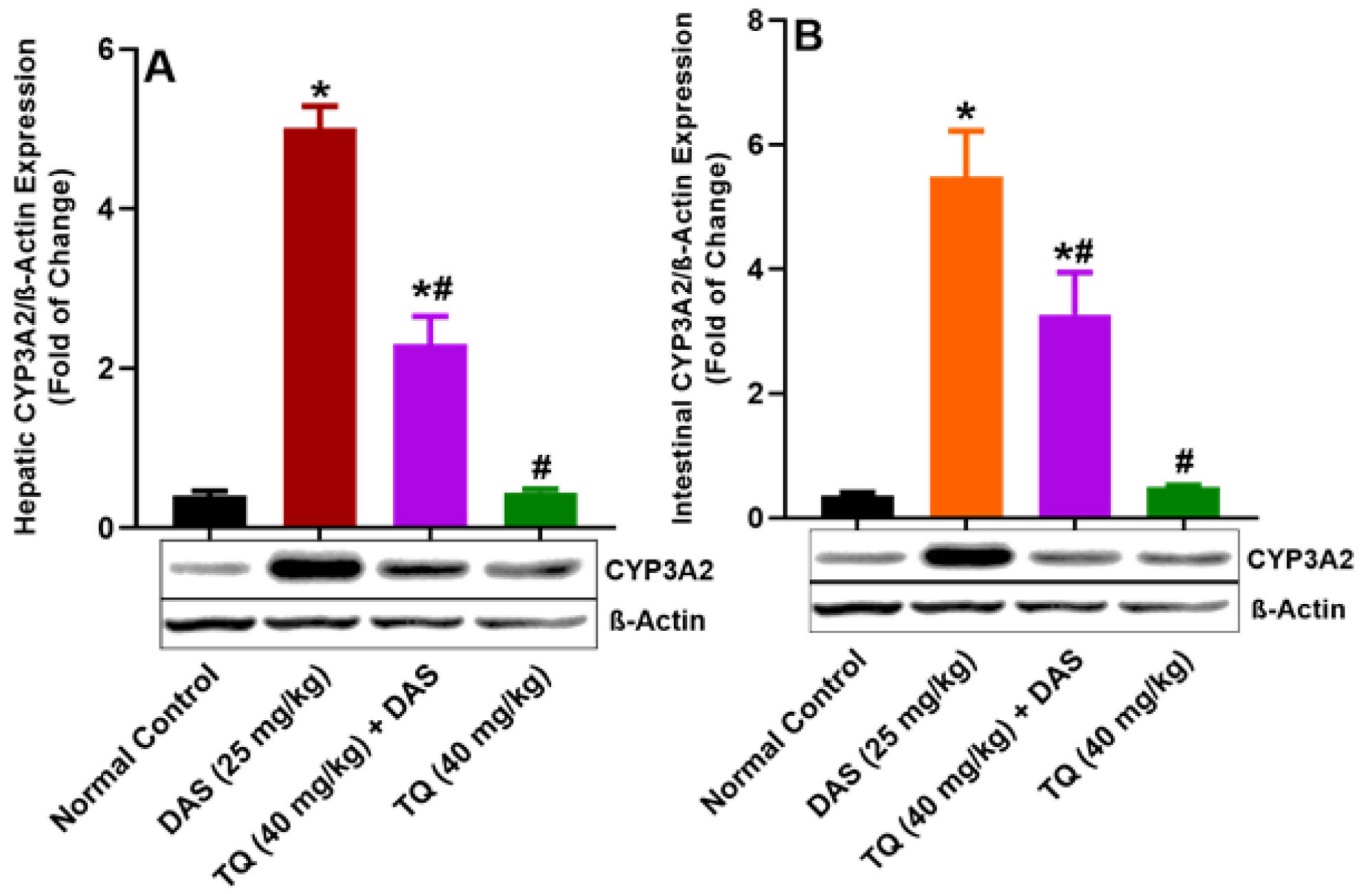
Figure illustrates the impact of TQ pretreatment on the expressions of CYP3A2, in the liver (A) and the intestinal lumen (B) of rats following dasatinib (DAS) administration. All data are presented as the mean ± SEM. * indicates *p<0.05* vs. normal controls; # indicates *p<0.05* vs. the DAS group (n= 6 per group).

As illustrated in Figure 3 (A, B), the protein expressions of CYP3A2 in the liver and lumen tissues was significantly elevated by 91.84% and 93.47%, respectively, following DAS administration to rats (*p<0.05*). However, pretreatment with TQ for seven days significantly suppressed this CYP3A2 upregulation by 53.98% in the liver and by 40.30% in the lumen, of rats which administered DAS. TQ alone also did not significantly altered CYP3A2 expressions as compared to normal controls.

**Figure 4:**
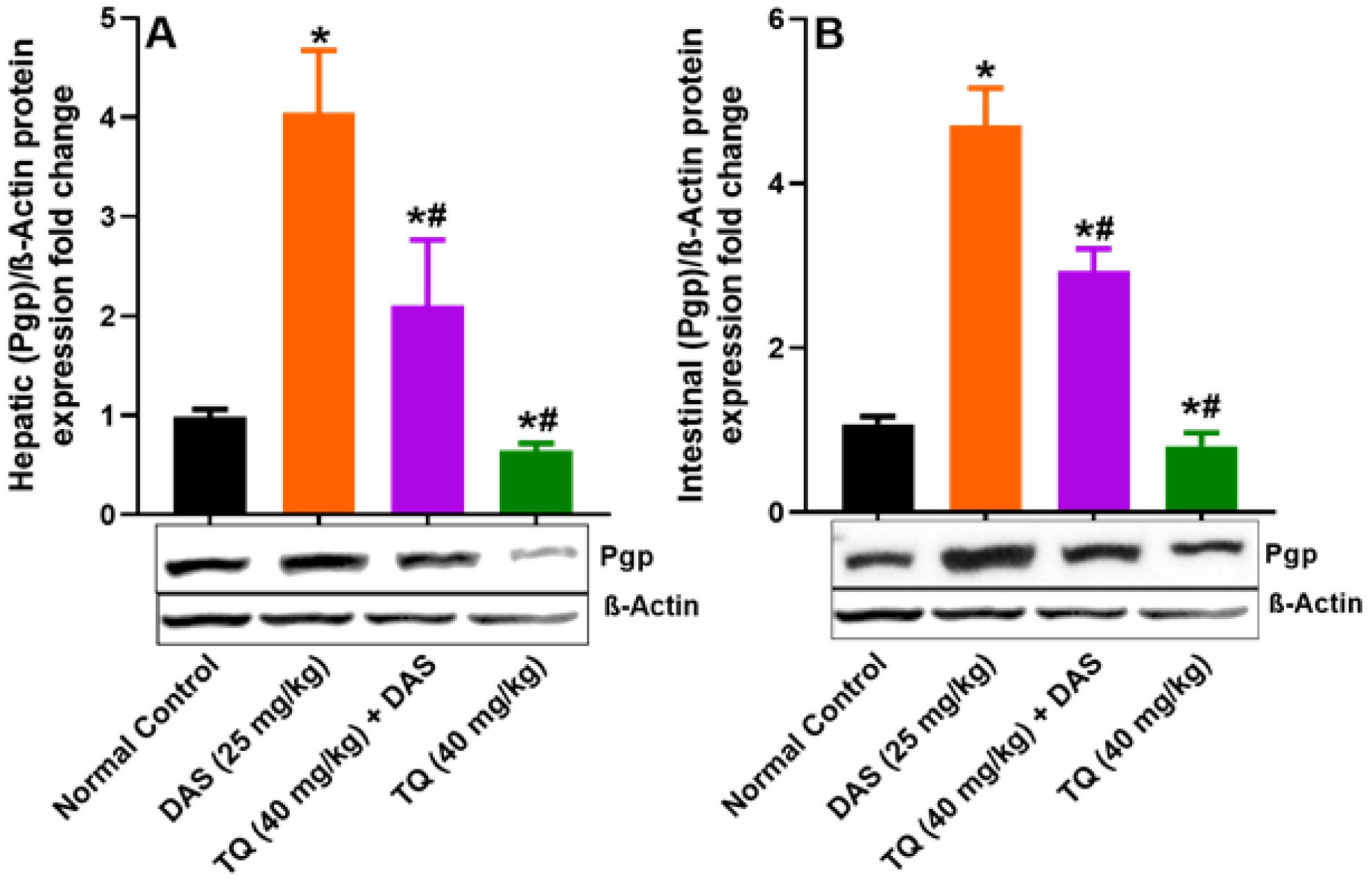
Figure illustrates the impact of TQ pretreatment on the expressions of Pgp, in the liver (A) and the intestinal lumen (B) of rats following dasatinib (DAS) administration. All data are presented as the mean ± SEM. * indicates *p<0.05* vs. normal controls; # indicates *p<0.05* vs. the DAS group (n= 6 per group).

Figure 4 (A, B) demonstrates the inhibitory effect of TQ on Pgp protein expression. Liver and lumen Pgp expressions were significantly increased by 75.62% and 77.19%, respectively (*p<0.05*), in rats treated with DAS compared to untreated animals. However, pretreatment with TQ significant suppressed the Pgp expressions in the liver and lumen tissues of rats treated with DAS by 47.97% and 37.72%, respectively (*p<0.05*). Similar to CYP3A2, TQ pretreatment alone did not affect the liver and lumen Pgp protein expressions compared to untreated animals.

**Figure 5:**
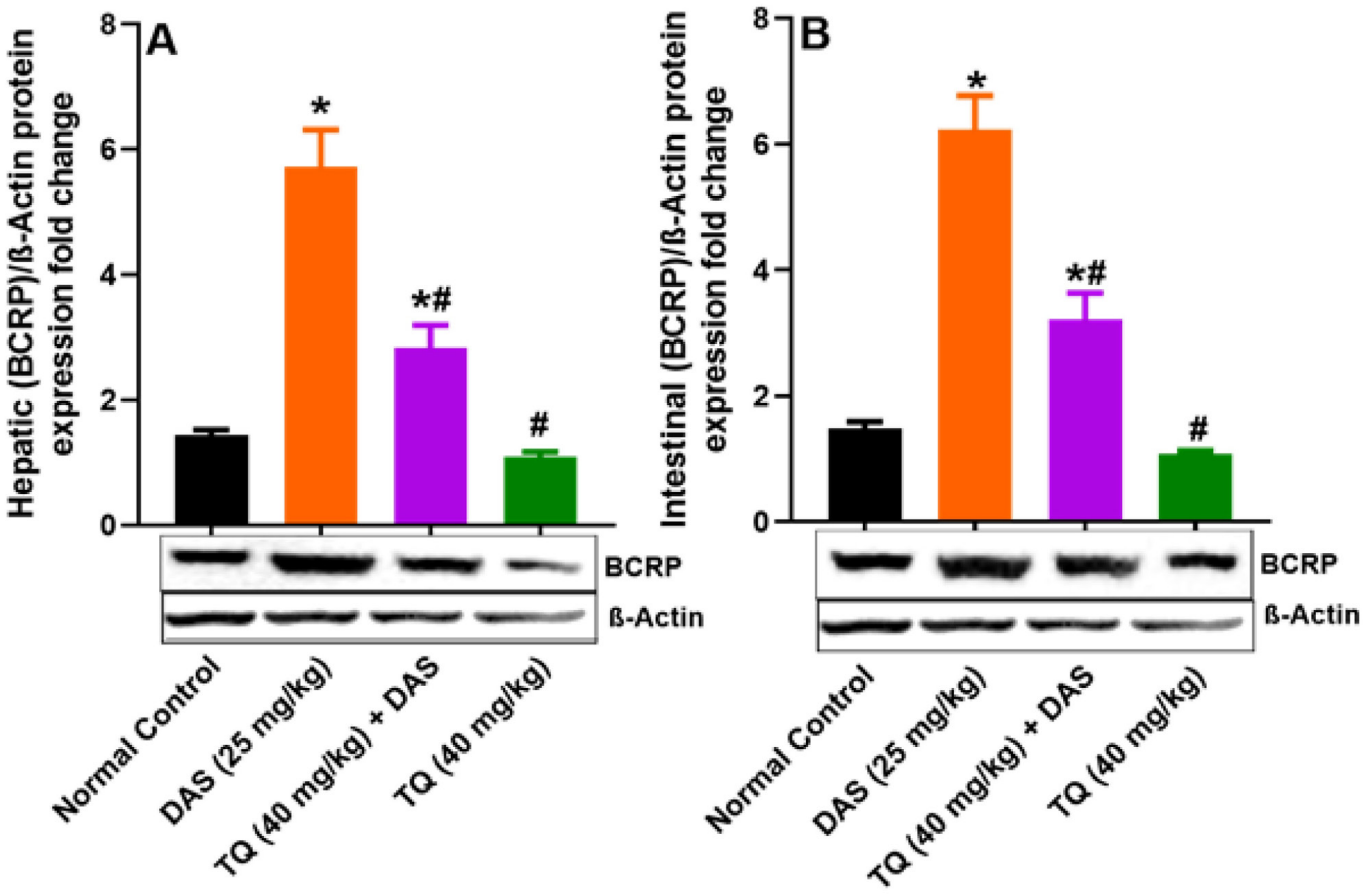
Figure illustrates the impact of TQ pretreatment on the expressions of BCRP in the liver (A) and the intestinal lumen (B) of rats following dasatinib (DAS) administration. All data are presented as the mean ± SEM. * indicates *p<0.05* vs. normal controls; # indicates *p<0.05* vs. the DAS group (n= 6 per group).

Figure 5 (A, B) presents the investigation into the potential inhibitory effect of TQ on BCRP protein expression, revealing no significant changes in the liver and lumen BCPR protein levels following TQ treatment compared to normal controls. Contrarily, oral administration of DAS resulted in a significant induction of BCRP protein expression in the liver and the lumen by 74.82% and 76.05%, respectively (*p<0.05*) as compared to untreated animals. Finally, TQ pretreatment in the DAS treatment group resulted in a significant inhibition of BCRP protein expression in the Liver by 50.48% and in the lumen tissues by 48.46% (*p<0.05*).

## Discussion

TKIs can be beneficial for the specific targeted treatment of an array of malignancies. The first medication to be employed in clinical oncology of this class of drugs was imatinib. Others such as inhibitorssunitinib, gefitinib, sorafenib, erlotinib, and dasatinib have emerged later 4. TKIs can induce toxicity to several organs, including the lungs, the kidneys, the heart, the liver, and the thyroid gland., In addition, blood coagulation, skin and nervous system reactions, and gastrointestinal tract adverse effects have been reported, irrespective of how well TKIs work as inhibitors targeting and translating this into therapeutic applications 36, 37. Cancer patients often use alternative therapies such as herbal and nutraceutical products and supplements, to enhance their general well-being and reduce the negative effects of chemotherapy 38, 39. The benefits of using such products are questionable due to the possibility of herb-drug interaction (HDI). The main mode that HDI work is by inhibiting or enhancing the expression of drug transporters and/or drug-metabolizing enzymes 40, 41. The benefits of using herbal products are questionable due to the possibility of herb-drug interaction (HDI). The main way that HDI interactions work is by inhibiting or enhancing the expression of drug transporters and/or drug-metabolizing enzymes (DME) 40, 41. DAS has been approved by FDA for use in patients with CML and Ph+ ALL who do not improve with first-line treatment. BCR-ABL and Src family kinases are its several targets that it inhibits 42. The CYP3A primarily metabolised DAS in liver, intestine and kidney. M4, M5, M6, M20, and M24 are the primary metabolites of DAS 9-11. Food substances have a wide range of interactions with proteins that are implicated in drug PK/PD profiles. These interactions can occur at any point from the absorption phase to potential changes in clinical efficacy, and they may also be related to pharmacological refractoriness, therapeutic side effects, or unanticipated adverse events. Considering that DAS is a substrate for CYP3A4, PGP/MDR1, and BCPR1 and has a narrow therapeutic spectrum 15, 17, 19, 35, 43-45. The concominant use of TQ along with prescribed drug DAS a potential pharmacokinetic interaction may occur the current investigation was proposed to verify whether or not TQ has a significant pharmacokinetic interaction with DAS, and also what possible mechanism(s) could be implicated. DAS has a dose-proportional pharmacokinetic profile and a linear elimination between 15 mg/day (0.15 times the lowest approved recommended dose) and 240 mg/day (1.7 times the highest approved recommended dose).The dose selected for DAS based on highest recommended dose human clinical dose that is 240 mg per day for adult (average weight 65 kg) is converted to rat dose which is 22.73 and acute toxicity studies demonstrated that 30 mg/kg DAS is well tolerated by rats therefore 25 mg/kg DAS selected for pharmacokinetic studies and it is within the limit.

Previous studies demonstrated administration of TQ to wistar rats of both sexes, the maximum tolerated doses (MTDs) for i.p. injection were 22.5 and 15 mg/kg in male and female rats, while MTD was 250 mg/kg in both male and female rats that received oral TQ. The dose selection was based on the basis of previous studies 46, 47. The previous studies reported therapeutic dose 48-51 hence selection of doses for DAS and TQ pharmacokinetic study are within the clinical limits. The pharmacokinetic study reveals that several fold increased in systemic exposure of DAS in TQ preteated animals as compared to DAS alone animals. TQ pretreatment or parallel administration of DAS (25 mg/kg) resulted in a substantial increase in the Cmax (ng/ml), AUC0-t (ng/(ml h), AUMC_0-∞_ (ng/(ml h), Kel (h^-1^) constant and Tmax and MRT, volumes of distribution Vd (mg/kg)/(ng/ml) and rate of clearance CL (mg/kg)/(ng/ml) of DAS (*p<0.05*) as compared to the normal control (DAS 25 mg/kg). These findings indicate that both systemic bioavailability and DAS exposures have risen. Most likely TQ, a strong inhibitor of drug-metabolizing enzymes (such as CYP3A4) and drug transporters, such as Pgp/MDR1 and BCRP1, may cause pharmacokinetic interactions. The literature shows that TQ inhibitors of CYP3A4 a drug metabolizing enzyme 28, 52-54 and drug transporters Pgp/MDR1 and BCRP1^55^-^58^. The pharmacokinetic parameters indicated an increase in the bioavailability of DAS as demonstrated by a rise in Cmax and AUC, and a reduction in its elimination as reflected by a decreased in T ½ (h) and CL. The increased in bioavailability as revealed by PK parameters may be due to suppression of CYP3A2, Pgp/MDR1, and BCPR/ABCG2-mediated dasatinib metabolism in the liver and intestine as demonstrated in the current study. The inhibition of ABCG2 and BCPR increased the absorption of drug and prevent elimination as evident by decreased T ½ (h) and CL. These results are in line with previous reports 45, 59-62. The prior studies conducted in our labs demonstrated that polyphenols (apigenin, sinapic acid and naringenin) have increased the systemic bioavailability of DAS 15, 17, 18, 35. HDI between TQ and DAS is likely due to TQ, a strong inhibitor of the CYPP3A2 and the drug transporters Pgp and BCRP 28, 52-54. Involvement of efflux transporters are evident as there is significant reduction in PK parameters MRT, T1/2, Vd and CL led to PK interaction that may cause accumulation of drug and inhibition of drug metabolism 14, 44, 45, 55.

The data of immunoblot experiments carried on the liver and lumen tissues demonstrated that TQ significantly reduced the upregulated CYP3A4, Pgp and BCRP protein expressions as compared to the DAS treatment group. This indicates that TQ has ability to modulate the expression of CYP3A2/Pgp/BCRP in the liver and lumen. This in accordance with increased DAS systemic bioavailability as evident by increased in its Cmax, Tmax, and AUC and decrease in Vd, MRT, CL of were evident in the TQ treatment group. TQ inhibits CYP3A2/Pgp/BCRP 28, Therefore, a thorough clinical investigation on the potential interaction between DAS and TQ-containing products is warranted for an appropriate regulation of the safety and effectiveness of drug therapy. Meanwhile, it is prudent to avoid the cocurrent use of TQ-containing products with DAS.

## Conclusion

The current study demonstrated that TQ pretreatment can significantly alters the disposition of DAS in an animal model. The increased in systemic bioavailability and is likely due to modulation of protein expression of CYP3A4 and Pgp and BCRP in the liver and lumen tissues. Thus, consuming TQ-containing food with DAS can lead to serious interactions and may pose a risk to patients. Furthermore, a clinical evaluation is needed to confirm such observations in humans.

## Figures and Tables

**Table 1:** The non-compartmental pharmacokinetic parameters for oral dasatinib (DAS, 25 mg/kg) with and without the co-administration of TQ, 40 mg/kg p.o. in rats (n= per group).

Pk Parameters	DAS	DAS+TQ	% Change
	Mean ±SEM	Mean ± SEM	
Kel (1/h)	0.09 ±0.01	0.18 ±0.01	93.85*
T_1/2_ (h)	7.77 ±0.65	3.95 ±0.21	-49.19*
Tmax (h)	1.00 ±0.00	1.83 ±0.17	83.33*
Cmax (ng/ml)	246.08 ±3.34	770.88 ±232.55	213.27*
AUC_0-t_ (ng/ml*h)	1614.93 ±28.86	4304.28 ±1289.08	166.53*
AUC_0-∞_ (ng/ml*h)	2501.79 ±112.63	4931.04 ±1507.76	97.10*
AUMC_0-∞_ (ng/ml*h^2^)	29259.52 ±3397.55	29359.54 ±9463.06	0.34*
MRT (h)	11.53 ±0.81	5.86 ±0.15	-49.13*
Vd (mg/kg)/(ng/ml)	0.11 ±0.00	0.04 ±0.004	-67.71*
CL (mg/kg)/(ng/ml)/h	0.01 ±0.00	0.01 ±0.001	-36.35

Data are presented as mean ±SEM. *indicates *p<0.05.*
